# Quasispecies Diversity and Amino Acid Variation at the Fusion Protein Cleavage Site of Newcastle Disease Virus Isolated from Cormorants in Central Asia

**DOI:** 10.3390/pathogens15101059

**Published:** 2026-10-08

**Authors:** Kobey Karamendin, Sardor Nuralibekov, Temirlan Sabyrzhan, Yermukhammet Kasymbekov, Klara Daulbayeva, Yelizaveta Khan, Marat Kumar, Aidyn Kydyrmanov

**Affiliations:** 1 Laboratory of Biochemistry of Viruses, Research and Production Center for Microbiology and Virology, 105 Bogenbay batyr str., Almaty 050010, Kazakhstan; 2 Laboratory of Viral Ecology, Research and Production Center for Microbiology and Virology, 105 Bogenbay batyr str., Almaty 050010, Kazakhstan; nuralibekovs@mail.ru (S.N.); zingyrin@gmail.com (T.S.); kasymbek.ermuxan@mail.ru (Y.K.); daulbaeva47@mail.ru (K.D.); lizaveta4ka@list.ru (Y.K.); kmarat882@gmail.com (M.K.); aidyn.kydyrmanov@gmail.com (A.K.)

**Keywords:** cormorant, Newcastle disease virus, quasispecies, cleavage site, RNA, F gene

## Abstract

Newcastle disease virus (NDV) remains a major threat to poultry production worldwide, and wild waterbirds are recognized as reservoirs of virulent strains. To characterize fine-scale quasispecies diversity at the virulence-determining fusion (F) protein cleavage site, amplicons encompassing the six-amino-acid motif were deep-sequenced from seven egg-passaged cormorant-derived isolates. Fifty-four distinct amino-acid substitutions passed predefined abundance and statistical filters and were detected across all six cleavage-site positions. The cleavage-site quasispecies was dominated by a limited set of recurrent substitutions (R1G, F6L, F6V, F6C, R4G, and several position-2 variants), each supported by hundreds of thousands of reads and occurring at frequencies of 1–26% across samples. Pooled Shannon entropy indicated that positions 1 and 6 were the most variable, whereas positions 3 and 5 showed lower diversity across the dataset. Principal component analysis showed apparent differences in mutation-frequency profiles among the seven egg-passaged isolates; the first two principal components explained 97.9% of the variance (PC1, 87.4%; PC2, 10.5%). Heatmap visualization showed that a subset of variants, including R1G and F6L/V/C, was consistently abundant across samples, whereas others were isolate-specific. These data describe position-specific cleavage-site heterogeneity in seven egg-passaged NDV isolates originally recovered from cormorants. The observed variant frequencies may reflect a combination of diversity present in the original specimens and changes introduced or selected during egg amplification. The coexistence of the predominant velogenic RRQRRF motif with lower-frequency alternative and stop-codon-containing haplotypes documents sequence heterogeneity in outbreak-related material after egg amplification and provides a descriptive basis for future genotype-resolved and phenotype-linked studies.

## 1. Introduction

Newcastle disease virus (NDV; *Avian orthoavulavirus 1*) is a highly contagious pathogen of poultry and wild birds that causes major economic losses worldwide [1]. It is an enveloped, single-stranded, negative-sense RNA virus of the family *Paramyxoviridae* with a genome of approximately 15.2 kb encoding six structural proteins [2,3].

NDV strains are classified as velogenic, mesogenic, or lentogenic according to their virulence in chickens, and this classification is strongly associated with the amino acid sequence at the cleavage site of the fusion precursor protein F0, a major determinant of virulence and tissue tropism [4]. The F protein is synthesized as an inactive precursor that must be cleaved into F1 and F2 to mediate membrane fusion and viral entry [5]. Lentogenic strains typically contain monobasic cleavage-site motifs (112-E(G)RQE(G)RL-117) that are processed only by proteases with restricted tissue distribution, limiting infection mainly to the respiratory and gastrointestinal tracts, whereas velogenic strains possess polybasic motifs (112-R(K)RQR(K)RF-117) recognized by ubiquitous furin-like proteases, enabling systemic infection [6].

RNA viruses exist within host organisms not as uniform populations of identical genomes but rather as complex distributions of closely related mutant variants, termed “quasispecies,” a concept originally developed by Manfred Eigen and Peter Schuster [7]. This phenomenon arises from the inherently high error rate of viral RNA-dependent RNA polymerase (RdRp), which lacks 3′ to 5′ exonuclease proofreading activity, resulting in mutation rates of approximately 10^−3^ to 10^−5^ nucleotide substitutions per site per replication cycle, several orders of magnitude higher than typical DNA-based replication systems [8]. The quasispecies structure represents a dynamic equilibrium between the continuous generation of mutant variants through error-prone replication and their elimination through negative selection based on fitness differences [9]. This mutant spectrum constitutes a major source of viral adaptability, providing phenotypic and genotypic diversity that enables viruses to rapidly adapt to changing environmental conditions, escape host immune responses, and develop antiviral resistance [10,11,12]. Recent advances in deep sequencing technologies have revealed complex quasispecies populations in NDV-infected hosts, with significant implications for understanding viral pathogenesis and emergence [13]. It was shown that lentogenic isolates can harbor low-frequency velogenic variants at the F-protein cleavage site, supporting the idea that virulence may emerge through selection of pre-existing minority variants rather than only through de novo mutation [14].

In this study, we used deep sequencing to characterize quasispecies diversity at the NDV F-protein cleavage site in seven isolates obtained from live infected cormorants and amplified in embryonated chicken eggs. By analyzing millions of aligned translated reads, we identified significant amino acid variants, compared their frequencies among isolates, assessed position-specific entropy, and evaluated clustering patterns among samples to characterize cleavage-site variability in outbreak-related material after egg amplification. This study is relevant to understanding the genetic processes that may occur when wild-type NDV enters and replicates in a susceptible host, particularly the emergence and persistence of genetic variation at the fusion-protein cleavage site, a region closely associated with viral entry, tissue tropism, and virulence.

## 2. Materials and Methods

Field samples were collected in June 2016 during the Newcastle disease outbreak among juvenile cormorants at Lake Alakol in Kazakhstan [15,16]. Samples underwent two serial passages in embryonated chicken eggs (blind and first passages). Hemagglutination assay-positive allantoic fluid samples from infected embryonated chicken eggs were used for RNA extraction using the QIAamp Viral RNA Mini kit (Qiagen, Hilden, Germany) according to the manufacturer’s protocol.

Reverse transcription-PCR assays were performed using the OneTaq One-Step RT-PCR Kit (New England BioLabs, Ipswich, MA, USA) according to the manufacturer’s instructions. PCR primers NDV-47-F ACACTCTTTCCCTACACGACGCTCTTCCGATCTATGGGCYCCARAYCTTCTAC and NDV-594-R GACTGGAGTTCAGACGTGTGCTCTTCCGATCTTGCATYTTYCCR ACTGCCAC targeting a fusion (F) gene fragment containing the cleavage site were designed with flanking Illumina sequencing adapters to facilitate direct library preparation. PCR amplification generated a defined fragment encompassing the six-amino-acid cleavage site region.

High-throughput libraries were constructed from PCR amplicons using the NEBNext Ultra DNA Library Preparation Kit (New England BioLabs) according to the manufacturer’s recommendations. Each amplicon library was assigned a unique sample-specific index during library preparation. Library quality was evaluated using a Bioanalyzer 2100 (Agilent Technologies, Santa Clara, CA, USA) with a High Sensitivity DNA Chip. Quantification was performed using Qubit 4 fluorometer (Invitrogen, Singapore). Paired-end sequencing (2 × 300 bp reads) was conducted on an Illumina MiSeq instrument using MiSeq Reagent Kit v3 (Illumina, San Diego, CA, USA) with 7.5% PhiX spike-in for sequence complexity assessment. Demultiplexing was performed using the instrument software according to the assigned sample indices, generating seven sample-specific FASTQ datasets corresponding to NDV-Quasi-1, -2, -4, -5, -6, -7, and -9. Raw sequencing data were processed and assembled using Geneious Prime 2025 (Dotmatics, Woburn, MA, USA) with standard quality-filtering parameters (minimum Phred quality score ≥ 30 for base calls). Obtained nucleotide sequences were translated and aligned to the RRQRRF (positions 1–6) reference sequence (GenBank accession #MK693033) using Geneious Prime 2025 with the MAFFT alignment algorithm.

All downstream analyses were performed in Python 3.10.12 using pandas (v2.3.3), numpy (v1.26.4), scipy (v1.15.3; scipy.stats.chi2_contingency), statsmodels (v0.14.5; statsmodels.stats.multitest.multipletests), matplotlib (v3.10.7), seaborn (v0.13.2), and scikit-learn (v1.7.2; sklearn.decomposition.PCA) on a Linux workstation. The script took as input the seven FASTA files of translated reads and output a table of mutation counts and statistics along with several plots. To determine whether substitutions at different cleavage-site positions co-occurred on the same sequencing read, complete six-residue haplotypes were extracted and quantified.

For each observed mutation, a chi-square test was performed [17] to determine whether the mutation frequency distributions differed significantly across the seven samples. The null hypothesis assumed that mutation frequencies would be equal across all samples. Statistical significance was evaluated at α = 0.05, with *p*-values adjusted for multiple comparisons using the Benjamini–Hochberg false discovery rate (FDR) method [18] to control the false discovery rate when analyzing mutations. *p*-values reported represent FDR-corrected values unless otherwise specified. All mutations with FDR-corrected *p*-values less than 0.05 were considered statistically significant.

A heatmap of log_10_ mutation frequencies was generated, with mutations as rows and samples as columns (white background, blue–yellow color scale). The heatmap was generated using standard bioinformatics visualization tools, with color intensity reflecting relative frequency [19,20]. Shannon entropy [21] was calculated at each of the six amino acid positions within the cleavage site region to quantify amino acid diversity across all samples combined. Principal component analysis (PCA) was performed on the matrix of mutation frequency profiles across samples to evaluate whether samples could be meaningfully clustered based on their mutational characteristics [22]. The two principal components, which explained the largest proportion of variance, were retained for visualization. PCA scores were plotted to reveal sample relationships and identify potential groupings based on shared patterns in mutation frequency. The 20 most abundant mutations were selected, and a bar chart was drawn showing total read counts per mutation (summed across all samples), with color gradients indicating rank.

## 3. Results

Analysis of seven FASTA files representing egg-passaged NDV isolates originally recovered from cormorants yielded millions of translated reads spanning the six amino acid positions corresponding to the F protein cleavage site, aligned with the reference sequence RRQRRF. The seven samples generated high-coverage sequencing data totaling approximately 13.7 million aligned reads (sample provenance, per sample read counts and mean depth are shown in Supplementary Table S1). This sequencing depth enables the detection of even rare variants within the quasispecies population and provides robust statistical power for comparative analysis across samples.

Chi-square analysis identified 54 distinct amino acid mutations at the cleavage site, all of which remained significant after FDR correction. The top 20 mutations by total read count showed that the quasispecies population was dominated by a limited subset of recurring variants (Figure 1A). The most abundant mutation was R1G at position 1, with 3,309,988 total reads and per-sample frequencies of 23–26%. At position 6, the substitutions F6L, F6V, and F6C each reached total counts of approximately 0.85–1.16 million reads and occurred at frequencies of about 6–9% per sample. R4G at position 4 was also consistently abundant, with approximately 400,555 total reads and per-sample frequencies of 2.7–3.3%. In addition, multiple substitutions at position 2, including R2G, R2*, R2S, R2I, R2K, R2V, R2T, and R2N, were detected at typical frequencies between 0.5% and 2%. Although RRQRRF remained the dominant consensus motif, each sample also contained a substantial fraction of reads with alternative single- or multi-residue variants.

Haplotype-level analysis showed that 48.3% of reads exactly matched the reference sequence RRQRRF, while 41.0% carried a substitution at exactly one of the six positions, together accounting for 89.4% of all reads. The remaining 10.6% of reads carried substitutions at two positions simultaneously, and a small fraction (0.12%) carried three co-occurring substitutions. The most abundant single-position variant was GRQRRF (position 1, R→G; 15.6% of reads), consistent with the marginal-frequency analysis. Among two-position haplotypes, the most abundant were GRQRRV and GRQRRL (both combining the position-1 R→G substitution with a position-6 substitution), together comprising 3.3% of reads. Stop-codon-containing haplotypes (e.g., RRQ*RF, R*QRRF, GRQ*RF) retained an otherwise intact basic-residue backbone at the remaining positions, with no haplotype observed in which a stop codon co-occurred with loss of more than one additional basic residue.

These findings indicate that the large majority of cleavage-site diversity in this dataset reflects independent, single-position substitutions overlaid on the polybasic RRQRRF backbone, while a smaller but consistent subset of reads (10.6%) represents genuine linked, multi-position haplotypes that could not have been resolved from marginal per-position frequencies alone. Classification of mutations by codon distance from the reference showed that double-nucleotide-distance substitutions were uniformly present at very low frequencies (≤0.27%), whereas single-nucleotide-distance substitutions dominated the dataset in both abundance and frequency range.

The mutation-frequency heatmap (Figure 1B) revealed both shared and sample-specific features of the cleavage-site quasispecies. Several mutations, particularly R1G, F6L, F6V, F6C, and R4G, were consistently abundant across all seven samples, indicating the presence of a common high-frequency mutational backbone. For instance, R1G shows log_10_-frequencies around −0.64 to −0.58 (corresponding to 23–26%), while F6L and F6V show log_10_-frequencies around −1.1 (≈8%). In contrast, some low-frequency substitutions were enriched in individual isolates, especially NDV-Quasi-4 and NDV-Quasi-9, consistent with sample-specific founder effects or within-host diversification. A positional gradient was also apparent: substitutions at positions 1, 2, 4, 5, and 6 were more prominent than those at position 3, which agrees with the lower entropy observed at that site. Overall, the heatmap suggests that several significant variants recur across samples rather than appearing as isolated sporadic events within the analyzed material.

The per-position Shannon entropy plot (Figure 1C) summarizes pooled amino-acid diversity across all reads from the seven egg-passaged isolates; it is not a within-bird measurement and does not independently demonstrate functional constraint or selection. Higher entropy values indicate greater diversity in the distribution of amino-acid residues at a given position. Positions 1 and 6 showed the greatest pooled diversity (approximately 1.07 and 1.34 bits, respectively), whereas positions 3 and 5 showed lower pooled diversity (approximately 0.35 and 0.26 bits, respectively).

PCA of mutation-frequency profiles (Figure 1D) was used as a descriptive visualization of apparent differences among the seven egg-passaged isolates. The first two principal components explained 97.9% of the variance in the unstandardized mutation-frequency matrix (PC1, 87.4%; PC2, 10.5%). NDV-Quasi-1, -5, and -6 grouped closely, whereas NDV-Quasi-9 was separated primarily along PC1; NDV-Quasi-2 and -4 showed separation along PC2. Given the small number of isolates and the absence of technical replicates, these patterns should be interpreted as descriptive differences in the observed mutation-frequency profiles rather than evidence of confirmed biological clustering.

## 4. Discussion

This study provides a high-resolution description of sequence heterogeneity at the NDV fusion-protein cleavage site in egg-passaged isolates originally recovered from cormorants in Central Asia. Because the original field specimens could not be sequenced, the resulting profiles cannot be assigned exclusively to viral populations replicating in cormorants and may have been influenced by bottlenecks or selection during embryonated-chicken-egg amplification. The NDV F-protein cleavage site is a major determinant of virulence: lentogenic strains typically contain monobasic motifs such as ERQERL, whereas velogenic strains harbor polybasic motifs such as RRQRRF that are recognized by furin-like proteases [23]. In our dataset, the velogenic motif was accompanied by numerous alternative variants, demonstrating substantial within-isolate diversity at this key virulence-associated region.

These findings are consistent with previous studies showing that positions R2, Q3, and R5 are relatively conserved between lentogenic and velogenic strains, whereas positions 1, 4, and 6 are more variable and contribute to motifs associated with different pathogenic phenotypes [13]. Accordingly, the most abundant substitutions detected here, including R1G, F6L/V/C, and R4G, were concentrated at these more permissive positions. Earlier work also showed that avirulent field isolates can harbor low-frequency virulent cleavage-site variants that may become enriched under selective pressure, and that velogenic viruses may emerge through amplification of pre-existing minority variants rather than only by de novo mutation [14]. Our results are consistent with the broader view of NDV as a genetically heterogeneous population in which minority variants at the cleavage site may be detected within a velogenic background.

The entropy analysis further emphasized positional differences in constraint. Positions 1 and 6 were the most variable, whereas positions 3 and 5 were comparatively conserved, consistent with prior evidence that not all residues within the cleavage motif contribute equally to protease recognition and fusion activation. The higher variability at positions 1 and 6 indicates greater tolerance for substitution. The intermediate diversity at position 4 is likewise consistent with the presence of multiple low-frequency R4 variants. Together, these patterns suggest that the cleavage site is functionally constrained but still permits limited sequence variation at selected positions.

The high frequency of R1G across all seven isolates indicates that this substitution was compatible with persistence in the analyzed egg-passaged material; however, its effect on F-protein cleavage, fusion activity, replication, or virulence was not tested. Likewise, the functional consequences of substitutions at position 6 remain unknown. Stop-codon-containing reads, such as R2* and R4*, would be expected to prevent production of a full-length functional F protein if they represent intact viral genomes. Such reads may therefore reflect defective viral genomes, defective templates, or technical artifacts rather than replication-competent virions.

The recurrence of R1G and other high-frequency minority variants across isolates indicates that these sequences were repeatedly detected after egg propagation, but the present cross-sectional dataset cannot determine their fate under continued passage. Their frequencies could increase if they confer a replication advantage in embryonated chicken eggs, remain stable through neutral processes, or decline if constrained by purifying selection or fitness trade-offs. Serial-passage experiments with sequencing at successive passage levels, combined with direct comparison of original field material and egg-passaged material, would be required to distinguish these alternatives.

PCA provided a descriptive visualization of apparent differences in mutation-frequency profiles among isolates, with NDV-Quasi-9 as the most separated sample. Although all isolates underwent the same nominal egg-passage scheme, these patterns cannot be attributed specifically to founding-population differences or stochastic drift: variation in the initial infectious dose, the number of effective replication cycles, embryo-to-embryo conditions, bottlenecks during propagation, and technical variation during extraction, reverse transcription, PCR, or library preparation could also have influenced variant frequencies. This study has several limitations. First, all viruses were analyzed after passage in embryonated chicken eggs, which may have influenced the relative abundance of minority variants. Second, the original cormorant clinical samples were not successfully reanalyzed because the archival viral RNA had degraded, preventing direct comparison between primary material and egg-passaged isolates. Therefore, we cannot exclude the possibility that some low-frequency variants, including lentogenic-like signatures, were selectively enriched during passage or were present as mixed viral populations in the original specimens. This study was limited to amplicon sequencing and did not include reverse-genetics, cell-based cleavage, fusion, replication, or pathogenicity assays; sequence variation cannot be interpreted as direct evidence of altered phenotype, virulence, or adaptive potential.

In any case, this study is generally informative regarding potential scenarios of infection in susceptible organisms with wild-type strains.

## 5. Conclusions

Deep sequencing of the NDV fusion-protein cleavage site in seven cormorant-derived isolates from Central Asia revealed extensive, position-specific quasispecies diversity superimposed on a velogenic RRQRRF consensus motif. Using a high-depth sequencing and computational workflow, the study identified a core set of abundant mutations, notably R1G and multiple F6 substitutions, and showed that their frequencies differed across samples. These findings document cleavage-site sequence heterogeneity in outbreak-related, cormorant-derived NDV isolates after egg amplification. Within the limits of the present dataset, the results provide a descriptive view of variation at a key virulence-associated motif and may help guide future genotype-resolved and phenotype-linked studies of NDV diversity in wild birds and poultry.

## Figures and Tables

**Figure 1:**
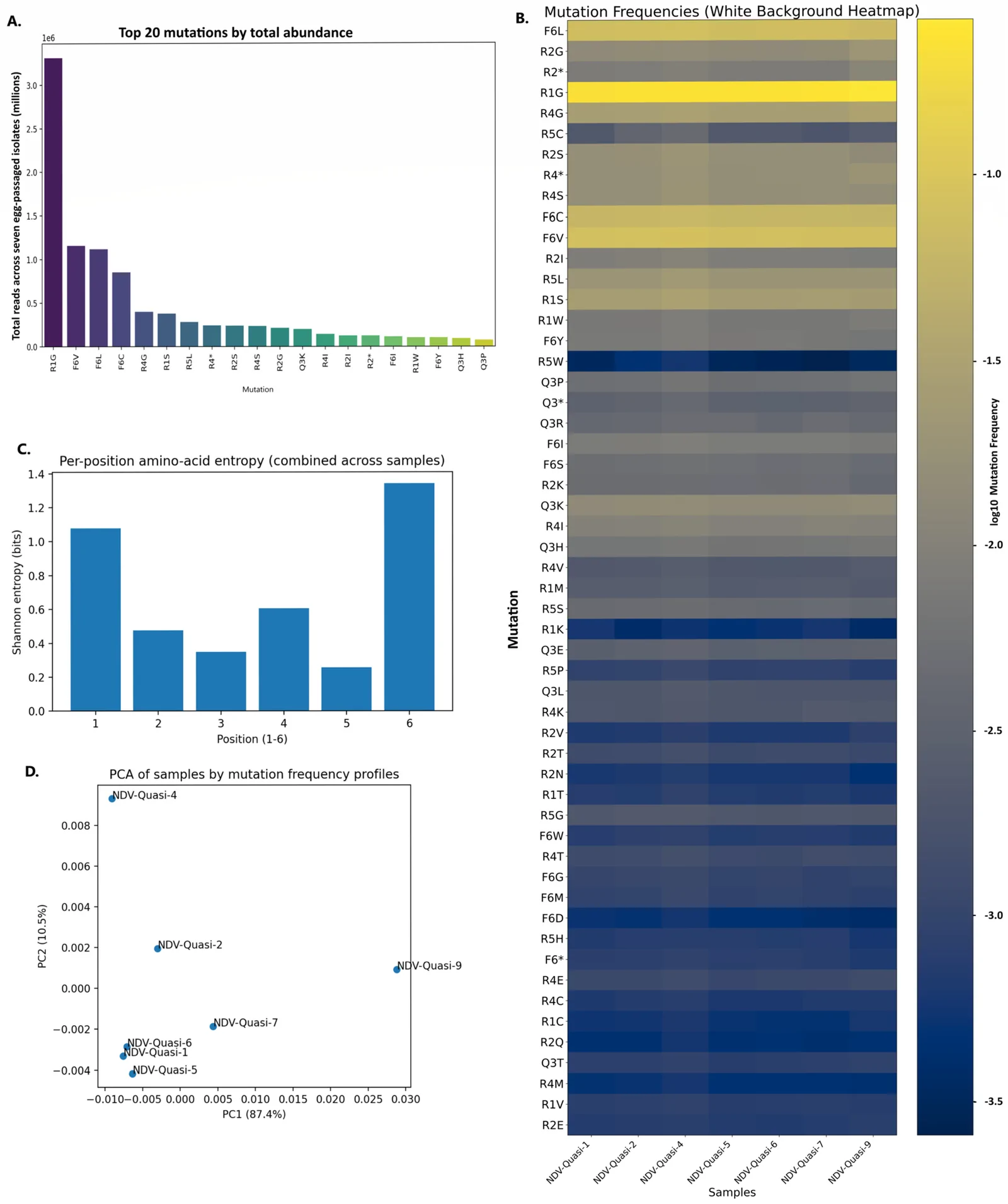
Plot of top 20 mutations (**A**). Heatmap of mutation frequencies (**B**), where warmer colors (yellow) represent higher frequencies, whereas cooler colors (dark blue) represent lower frequencies; per-position Shannon entropy (**C**), calculated from pooled reads across all seven egg-passaged isolates. It is a descriptive measure of amino-acid diversity: higher values indicate a more diverse distribution of residues at a position, whereas a value of zero would indicate complete conservation; and principal component analysis (**D**).

## Data Availability

The dataset generated for this study is available in the GenBank database under accession number PRJNA1523016.

## Supplementary Materials

The following supporting information can be downloaded at: https://www.mdpi.com/article/10.3390/pathogens15101059/s1, Table S1: Sample provenance, sequencing coverage, and passage history.

## Abbreviations

The following abbreviations are used in this manuscript:

NDV	Newcastle disease virus
PCA	Directory of open access journals
FDR	Three-letter acronym

## References

[1] Karamendin K., Kydyrmanov A., Fereidouni S., Fereidouni S. (2024). Avian Paramyxoviruses in Wild Birds. Ecology of Wild Bird Diseases.

[2] Alexander D.J. (2000). Newcastle disease and other avian paramyxoviruses. Rev. Sci. Tech.-Off. Int. Epizoot..

[3] Diel D.G., da Silva L.H., Liu H., Wang Z., Miller P.J., Afonso C.L. (2012). Genetic diversity of avian paramyxovirus type 1: Proposal for a unified nomenclature and classification system of Newcastle disease virus genotypes. Infect. Genet. Evol..

[4] Kondakova O.A., Agranovsky A.A., Ryabchevskaya E.M., Umarova E.P., Granovskiy D.L., Toropov S.E., Evtushenko E.A., Nikitin N.A., Karpova O.V. (2025). Genetic Diversity of Newcastle Disease Virus and Its Implications for Vaccine Development. Vet. Sci..

[5] Alexander D.J. (2011). Newcastle disease in the European Union 2000 to 2009. Avian Pathol..

[6] Collins M.S., Bashiruddin J.B., Alexander D.J. (1993). Deduced Amino-Acid-Sequences at the Fusion Protein Cleavage Site of Newcastle-Disease Viruses Showing Variation in Antigenicity and Pathogenicity. Arch. Virol..

[7] Andino R., Domingo E. (2015). Viral quasispecies. Virology.

[8] Lauring A.S., Andino R. (2010). Quasispecies theory and the behavior of RNA viruses. PLoS Pathog..

[9] Domingo E., Sheldon J., Perales C. (2012). Viral quasispecies evolution. Microbiol. Mol. Biol. Rev..

[10] Domingo E. (2002). Quasispecies Theory in Virology. J. Virol..

[11] Vignuzzi M., Stone J.K., Arnold J.J., Cameron C.E., Andino R. (2006). Quasispecies diversity determines pathogenesis through cooperative interactions in a viral population. Nature.

[12] Wilke C.O., Wang J.L., Ofria C., Lenski R.E., Adami C. (2001). Evolution of digital organisms at high mutation rates leads to survival of the flattest. Nature.

[13] Meng C., Qiu X., Yu S., Li C., Sun Y., Chen Z., Liu K., Zhang X., Tan L., Song C. (2016). Evolution of Newcastle Disease Virus Quasispecies Diversity and Enhanced Virulence after Passage through Chicken Air Sacs. J. Virol..

[14] Kattenbelt J.A., Stevens M.P., Selleck P.W., Gould A.R. (2010). Analysis of Newcastle disease virus quasispecies and factors affecting the emergence of virulent virus. Arch. Virol..

[15] Karamendin K., Kydyrmanov A., Kasymbekov Y., Daulbayeva K., Khan E., Seidalina A., Sayatov M., Gavrilov A., Fereidouni S. (2019). Cormorants as Potential Victims and Reservoirs of Velogenic Newcastle Disease Virus (Orthoavulavirus-1) in Central Asia. Avian Dis..

[16] Karamendin K., Kydyrmanov A. (2021). Cormorants as a Potentially Important Reservoir and Carrier of Newcastle Disease Virus on the Asian Continent. Front. Vet. Sci..

[17] Virtanen P., Gommers R., Oliphant T.E., Haberland M., Reddy T., Cournapeau D., Burovski E., Peterson P., Weckesser W., Bright J. (2020). SciPy 1.0: Fundamental algorithms for scientific computing in Python. Nat. Methods.

[18] Benjamini Y., Hochberg Y. (1995). Controlling the False Discovery Rate—A Practical and Powerful Approach to Multiple Testing. J. R. Stat. Soc. Ser. B-Stat. Methodol..

[19] Hunter J.D. (2007). Matplotlib: A 2D graphics environment. Comput. Sci. Eng..

[20] Waskom M.L. (2021). Seaborn: Statistical data visualization. J. Open Source Softw..

[21] Shannon C.E. (1997). The mathematical theory of communication. 1963. MD Comput..

[22] Pedregosa F., Varoquaux G., Gramfort A., Michel V., Thirion B., Grisel O., Blondel M., Prettenhofer P., Weiss R., Dubourg V. (2011). Scikit-learn: Machine Learning in Python. J. Mach. Learn. Res..

[23] Jadhav A., Zhao L., Liu W., Ding C., Nair V., Ramos-Onsins S.E., Ferretti L. (2020). Genomic Diversity and Evolution of Quasispecies in Newcastle Disease Virus Infections. Viruses.

