# Supplementary material for: Quasispecies Diversity and Amino Acid Variation at the Fusion Protein Cleavage Site of Newcastle Disease Virus Isolated from Cormorants in Central Asia
Source: Pathogens. 2026 Oct 8;15(10):1059. doi: 10.3390/pathogens15101059 (PMC13708561; doi:10.3390/pathogens15101059)
Supplement: Supplementary file 1 [file pathogens-15-01059-s001.zip › pathogens-4590356-supplementary.pdf]

Table S1. Sample provenance, sequencing coverage, and passage history

Sample	Bird ID	Specimen	Collection date	Passage history	Reads total	Coverage
NDV-Quasi-1	APMV-1/53/2016	Cloacal swab	16 June 2016	Two passages in embryonated chicken eggs	2,351,276	2,087,917
NDV-Quasi-2	APMV-1/58/2016	Cloacal swab	16 June 2016	Two passages in embryonated chicken eggs	2,077,470	1,801,893
NDV-Quasi-4	APMV-1/63/2016	Cloacal swab	16 June 2016	Two passages in embryonated chicken eggs	1,767,343	1,494,531
NDV-Quasi-5	APMV-1/69/2016	Cloacal swab	16 June 2016	Two passages in embryonated chicken eggs	2,759,786	2,428,090
NDV-Quasi-6	APMV-1/71/2016	Cloacal swab	16 June 2016	Two passages in embryonated chicken eggs	2,181,406	1,924,529
NDV-Quasi-7	APMV-1/73/2016	Cloacal swab	16 June 2016	Two passages in embryonated chicken eggs	2,792,307	2,398,949
NDV-Quasi-9	APMV-1/88/2016	Cloacal swab	16 June 2016	Two passages in embryonated chicken eggs	2,019,787	1,753,524
